# HIV-associated painful neuropathy: where are we?

**DOI:** 10.1590/0004-282X-ANP-2021-0079

**Published:** 2022-08-08

**Authors:** Karina Lebeis Pires, Marcelle Seraphim de Menezes Baranda, Talita Mota Almeida Brum, Bruno Pessôa, Camila Castelo Branco Pupe, Osvaldo José Moreira do Nascimento

**Affiliations:** 1 Universidade Federal Fluminense, Departamento de Neurologia, Unidade de Pesquisa Clínica, Niterói RJ, Brazil.; 2 Universidade Federal Fluminense, Departamento de Neurocirurgia, Unidade de Pesquisa Clínica, Niterói RJ, Brazil.; 3 Universidade Federal do Estado do Rio de Janeiro, Departamento de Neurologia, Unidade de Pesquisa Clínica, Niterói RJ, Brazil.

**Keywords:** Morbidity, HIV, Quality of Life, Morbidade, HIV, Qualidade de Vida

## Abstract

**Background:**

After the advent of combination antiretroviral therapy, infection with the human immunodeficiency virus (HIV) ceased to be a devastating disease, but sensory neuropathy resulting from the permanence of the virus and the side effects of treatment have worsened the morbidities of these patients.

**Objective:**

To investigate the quality of life of 64 HIV-positive patients: 24 with painful neuropathy (case group) and 40 without painful neuropathy (control group). The impact of other factors on quality of life was also assessed.

**Methods:**

To assess painful neuropathy, the Leeds Assessment of Neuropathic Symptoms and Signs (LANSS) scale, Douleur Neuropathique 4 (DN4) questions and Neuropathy Disability Score (NDS) were used. The Short Form Health Survey (SF-36) scale was used to assess quality of life. Factors related or unrelated to HIV were obtained through the medical history and analysis on medical records.

**Results:**

The quality of life of patients with neuropathic pain was worse in six of the eight domains of the SF-36 scale. The number of clinical manifestations related to HIV, length of time with detectable viral load since diagnosis, length of time since the diagnosis of HIV infection and length of time of HAART use had a negative impact on quality of life. Higher levels of CD4, education and family income had a positive impact.

**Conclusions:**

Painful neuropathy related to HIV is a factor that worsens the quality of life of patients infected with this virus and should be included in the clinical evaluation.

## RESUMO

**Antecedentes:**

Após o advento da terapia antirretroviral combinada a infecção pelo vírus da imunodeficiência humana (HIV) deixou de ser uma doença devastadora, porém a neuropatia sensitiva consequente à permanência do vírus e ao efeito colateral do tratamento piora a morbidade desses pacientes.

**Objetivo:**

Investigar a qualidade de vida de 64 pacientes com HIV, 24 com neuropatia dolorosa (grupo caso) e 40 sem neuropatia dolorosa (grupo controle). Avaliou-se também o impacto de outros fatores relacionados e não relacionados ao HIV na qualidade de vida.

**Métodos:**

Para avaliação da neuropatia dolorosa foram utilizadas as escalas *Leeds Assessment of Neuropathic Symptoms and Signs* (LANSS), *Douleur Neuropathique 4* (DN4) e Escore de Comprometimento Neuropático (ECN). Para avaliação da qualidade de vida foi utilizada a escala *Short Form Health Survey* (SF-36). Fatores relacionados e não relacionados ao HIV foram obtidos através da anamnese e análise de prontuário.

**Resultados:**

A qualidade de vida dos pacientes com dor neuropática foi pior em 6 dos 8 domínios da escala SF-36. O número de manifestações clínicas relacionadas ao HIV, tempo de carga viral detectável desde o diagnóstico, tempo de diagnóstico da infecção pelo vírus e tempo de uso de TARVC impactaram negativamente na qualidade de vida. Maior nível de CD4, da escolaridade e da renda familiar impactaram positivamente.

**Conclusões:**

A neuropatia dolorosa relacionada ao HIV é fator de piora da qualidade de vida dos pacientes infectados por esse vírus devendo ser incluída na avaliação clínica desses pacientes.

## INTRODUCTION

Infection by the human immunodeficiency virus (HIV) has been challenging healthcare authorities since the 1980s. In Brazil, 882,810 cases of acquired immunodeficiency syndrome (AIDS) were identified between 1980 and June 2017^1^. In 2014, with the inclusion of HIV infection in the list of compulsorily notifiable diseases, better understanding of the epidemiological profile of HIV/AIDS cases was achieved, thus enabling redirection of public healthcare policies. Many advances have been observed over recent years with regard to diagnosis and treatment of infection by this virus, but this remains an important public health problem.

In 2015, mortality due to HIV/AIDS was 42.3% lower than in 1995^1^. Neurological impairment has a major impact on both morbidity and mortality. It has been estimated that 40 to 70% of these patients have both central and peripheral neurological manifestations^2^. With the advent of antiretroviral therapy (ART), there was a reduction in mortality, but the length of exposure to the virus and to ART increased. Thus, there was a reduction in neurological impairment secondary to opportunistic infections related to severe immunodepression states. In contrast, the symptoms associated with the permanence of the virus and exposure to ART, such as peripheral neuropathy, remain the main neurological changes^2^ ^-^ ^5^. 

The incidence rate for sensory neuropathy (SN), and particularly distal symmetric polyneuropathy (DSP), ranges from 30 to 60% among HIV-positive patients^5^. SN has been found to be the most frequent form of neuropathy among HIV-positive individuals^6^ ^-^ ^8^. Its clinical manifestations are variable but include burning pain, numbness and paresthesia, which can be disabling and irreversible, with an important impact on quality of life^9^ ^,^ ^10^ . 

Some studies have assessed the influence of factors either related or unrelated to infection, on quality of life^11^ ^,^ ^12^. However, these studies did not assess the impact of neuropathic pain on the quality of life of HIV-positive patients. The aim of the present study was to investigate the quality of life of HIV-infected patients with neuropathic pain.

## METHODS

This study was approved by the Research Ethics Committee of Federal Fluminense University. Written informed consent was obtained from all study participants before enrollment into the study.

This was an observational and descriptive cross-sectional study. A convenience sample of adult HIV-infected patients either with or without neuropathic pain was evaluated between March and July 2017. Right after their consultations at the HIV/AIDS immunology clinic, patients who presented with pain in the lower limbs and/or upper limbs were referred for neurological evaluation. In this evaluation, anamnesis, physical examination and application of three neuropathic pain scales were used to characterize whether the referred pain was neuropathic or not. These scales were the Leeds Assessment of Neuropathic Symptoms and Signs (LANSS) scale, Douleur Neuropathique 4 (DN4) questions and Neuropathy Disability Score (NDS).

Next, a review of the medical records of patients with neuropathic pain was carried out to collect test results and epidemiological and disease-related data. Patients who had neuropathic pain on the three pain scales and who met the inclusion criteria were evaluated regarding quality of life (SF-36 scale). Patients without any type of pain, and with sociodemographic characteristics similar to those of the group with neuropathic pain, were selected as a control group to be evaluated using the SF-36 scale.

The inclusion criteria for both groups were that the participants needed to be between the ages of 18 and 65 years; have a positive HIV test on the blood sample; have complementary test results within the normal range, including normal TSH, FT4, B12 vitamin, cholesterol and glycated hemoglobin; and have no history of treatment for HIV/AIDS.

For the HIV-positive group with neuropathic pain, the additional inclusion criteria were that the participants needed to present the following; sensory neuropathic symptoms such as paresthesia, numbness and pain in the extremities; the criteria for neuropathic pain of two scales: LANSS ≥ 12 and DN4 ≥ 4; neuropathy on the clinical scale (NDS > 3); and a neurological examination compatible with peripheral neuropathy. 

For the HIV-positive control group, there was the additional inclusion criterion of not presenting any neurological symptoms. 

The exclusion criteria for both groups were the following situations: presence of an opportunistic disease that was either active or under treatment; presence of cognitive deficits, as assessed using the Mini-Mental State Examination (MMSE), with a score lower than 20^11^; presence of sensory deficits that prevented scoring of scales or that impaired test performance; and/or a history of drug-induced neuropathy.

For the control group, there was one further exclusion criterion: use of medication with action against neuropathic pain.

### Statistical analysis

Fisher's exact test was used to assess statistical significance between categorical variables, and between groups with and without painful neuropathy. Continuous variables were categorized so that statistical associations could be assessed using Fisher's exact test. Continuous variables were described in terms of means, medians and standard deviations using the nonparametric Mann-Whitney test. Statistically significant differences regarding the domains of the SF-36 scale, for groups with and without painful neuropathy, were calculated using the Mann-Whitney nonparametric test. The magnitude of differences between the means of the groups with and without painful neuropathy, in relation to each domain of the SF-36 scale, was evaluated by calculating Cohen's effect size. Statistical correlations within each group, between the domains of the SF-36 scale and the continuous and categorical variables, were performed using the Spearman correlation coefficient (rs) and the Mann-Whitney method, respectively. The statistical procedures were performed using the Statistical Package for the Social Sciences software, version 16.0.

### Description of the variables analyzed

The patients were divided into two groups (with and without neuropathy), and the variables analyzed were: sex, age, education, family income, practicing of physical activity, history of depression, BMI (body mass index), viral load (detectable or undetectable), duration of HIV infection, number of ARV drugs used, length of ART use, number of clinical manifestations related to HIV, CD4 value in the last six months and length of time for which a viral load (VL) had been detectable since diagnosis.

## RESULTS

Sixty-four patients were evaluated, among whom 24 presented painful neuropathy in accordance with the clinical criteria. The other 40 patients did not present painful neuropathy through the same criteria and thus composed the control group. In the group with painful neuropathy, 54.2% were female, 50% were between 40 and 49 years old, 41.7% had attended school for up to 5 years and 45.8% had a family income of 3 to 4 minimum monthly wages. In the control group, 55% were female, the predominant age group was 31 to 49 years (70%), the schooling level was higher in this group than in the other group (72.5% of the patients were aged 6 to 9 years of schooling) vs 41.7% with up to 5 years of schooling in the other group and 50% had a family income of between 3 and 4 minimum monthly wages. Table 1 characterizes the sociodemographic variables in relation to the two groups studied.

**Table 1.** Sociodemographic characteristics of the groups with and without neuropathy.

Sociodemographic variables		HIV-positive patients				P-value of Fisher's exact test
		WITH painful neuropathy (n = 24)		NO painful neuropathy (n = 40)		
		n	%	n	%	
Sex	Female	13	54.2	22	55.0	1.000
	Male	11	45.8	18	45.0	
Age	31 - 39 years	5	20.8	14	35.0	0.396
	40 - 49 years	12	50.0	14	35.0	
	50 - 55 years	7	29.2	12	30.0	
Educational level	Up to 5 years of schooling	10	41.7	9	22.5	0.001
	6 - 9 years of schooling	7	29.2	29	72.5	
	10 or more years of schooling	7	29.2	2	5.0	
Income (minimum monthly wages)	1 - 2 mw	3	12.5	1	2.5	0.353
	2 - 3 mw	9	37.5	14	35.0	
	3 - 4 mw	11	45.8	20	50.0	
	> 4 mw	1	4.2	5	12.5	

Source: HUGG HIV/AIDS immunology outpatient clinic; mw: minimum monthly wages.

Only the educational level variable showed a statistically significant difference (p-value < 0.05) between the groups, such that lower levels of education were concentrated in the group with neuropathy. There was a predominance of patients between 40 and 49 years old in the group with neuropathy, thus suggesting that increasing age may be related to a greater chance of developing neuropathy.

### Comparison of non-HIV-related variables between the groups with and without neuropathy

Considering the variables not related to HIV (Table 2), there was no statistically significant difference between the groups regarding the practice of physical activity, or regarding histories of depression. Despite this, 54.2% had a history of depression, as opposed to 47.5% in the control group. BMI showed a statistically significant difference, such that overweight predominated in the group with neuropathy.

**Table 2.** HIV-positive patients with and without painful neuropathy, according to variables not related to HIV.

Variables not related to HIV		HIV+ patients				P-value of Fisher's exact test
		WITH painful neuropathy (n = 24)		NO painful neuropathy (n=40)		
		n	%	n	%	
Physical activity	Yes	5	20.8	16	40.0	0.170
	No	19	79.2	24	60.0	
BMI (kg/m^2^)	Up to 24.9 (normal)	7	29.2	25	62.5	0.003
	25 - 29.9 (overweight)	14	58.3	7	17.5	
	30 or more (obesity)	3	12.5	8	20.0	
History of depression	Yes	13	54.2	19	47.5	0.797
	No	11	45.8	21	52.5	

Source: HUGG HIV/AIDS immunology outpatient clinic; BMI: body mass index.

### Comparison of HIV-related variables between the groups with and without painful neuropathy

The analysis of variables related to HIV is shown in Table 3. The only variable that demonstrated statistical significance between the groups was the length of time with a diagnosis of HIV infection. In the group without painful neuropathy, 60% of the patients had had this infection for less than 10 years. In the group with painful neuropathy, 58.4% of the patients had had it for more than 10 years.

**Table 3.** Patients with and without painful neuropathy, according to variables related to HIV.

Variables related to HIV		HIV-positive patients				P-value of Fisher's exact test
		WITH painful neuropathy (n = 24)		NO painful neuropathy (n=40)		
		n	%	n	%	
Length of time with the diagnosis of HIV infection	Up to 4 years	5	20.8	3	7.5	0.006
	5 - 9 years	5	20.8	21	52.5	
	10 - 14 years	7	29.2	14	35.0	
	15 or more	7	29.2	2	5.0	
Number of ARV used	Up to 5	18	75.0	34	85.0	0.341
	6 or more	6	25.0	6	15.0	
Length of HAART use	Up to 9 years	11	45.8	26	65.0	0.192
	10 or more	13	54.2	14	35.0	
Number of hospitalizations	0	9	37.5	23	57.5	0.071
	1	6	25.0	12	30.0	
	2 or more	9	37.5	5	12.5	
Number of clinical manifestations related to HIV	0	3	12.5	14	35.0	0.130
	1	9	37.5	13	32.5	
	2 or more	12	50.0	13	32.5	
History of abandonment of HAART use	Yes	8	33.3	14	35.0	1.000
	No	16	66.7	26	65.0	
CD4 cell count	Up to 499	6	25.0	13	32.5	0.583
	500 or more	18	75.0	27	67.5	
Viral load	Undetectable	20	83.3	35	87.5	0.718
	Detectable	4	16.7	5	12.5	
Time of VL detectable since diagnosis	1 year	8	33.3	26	65.0	0.051
	2 years	7	29.2	6	15.0	
	3 years or more	9	37.5	8	20.0	

Source: HUGG HIV/AIDS immunology outpatient clinic; ARV: antiretrovirals; HAART: highly active antiretroviral therapy; VL: viral load.

### Analysis of numerical variables using the Mann-Whitney test

Descriptive statistics on numerical variables among the HIV-positive patients with and without painful neuropathy are presented in Table 4. Only the variables of the number of clinical manifestations and the length of time with detectable viral load since diagnosis showed statistically significant differences between the groups studied, such that they were greater in the group with neuropathy.

**Table 4.** Descriptive statistics for numerical variables among HIV-positive patients with and without painful neuropathy.

Variables	HIV-positive groups	Descriptive statistics					Mann-Whitney test p-value
		Average	Standard deviation	Minimum	Median	Maximum	
Age (years)	With painful neuropathy	46.7	5.8	35.0	47.5	55.0	0.296
	No painful neuropathy	44.2	7.4	31.0	43.5	55.0	
Years of study	With painful neuropathy	6.7	3.0	4.0	6.0	13.0	0.583
	No painful neuropathy	6.6	1.7	4.0	6.0	12.0	
Income (MW)	With painful neuropathy	2.4	0.8	1.0	2.5	4.0	0.155
	No painful neuropathy	2.7	0.7	1.0	3.0	4.0	
BMI (kg/m^2^)	With painful neuropathy	26.2	5.4	18.3	26.0	42.3	0.291
	No painful neuropathy	25.7	6.1	18.0	24.7	40.0	
Infection duration (years)	With painful neuropathy	10.7	5.5	2.0	11.5	21.0	0.117
	No painful neuropathy	8.7	4.0	4.0	8.0	21.0	
Number of ARV used	With painful neuropathy	4.5	1.5	3.0	4.0	9.0	0.522
	No painful neuropathy	4.2	1.2	3.0	4.0	7.0	
Duration of use of HAART (years)	With painful neuropathy	9.3	4.8	2.0	10.5	19.0	0.185
	No painful neuropathy	7.8	3.1	4.0	7.0	14.0	
Number of clinical manifestations	With painful neuropathy	1.8	1.3	0.0	1.5	4.0	0.026
	No painful neuropathy	1.1	1.1	0.0	1.0	5.0	
CD4	With painful neuropathy	670	345	14	682	1.257	0.321
	No painful neuropathy	760	313	221	814	1.327	
Detectable VL duration (years)	With painful neuropathy	2.9	2.7	1.0	2.0	12.0	0.019
	No painful neuropathy	1.9	1.6	1.0	1.0	8.0	

Source: HUGG HIV/AIDS immunology outpatient clinic. MW: minimum monthly wage; BMI: body mass index; ARV: antiretrovirals; HAART: highly active antiretroviral therapy; VL: viral load.

### Differentiation of the groups with and without neuropathy in relation to the domains of the SF-36 quality-of-life scale

**Figure 1.**
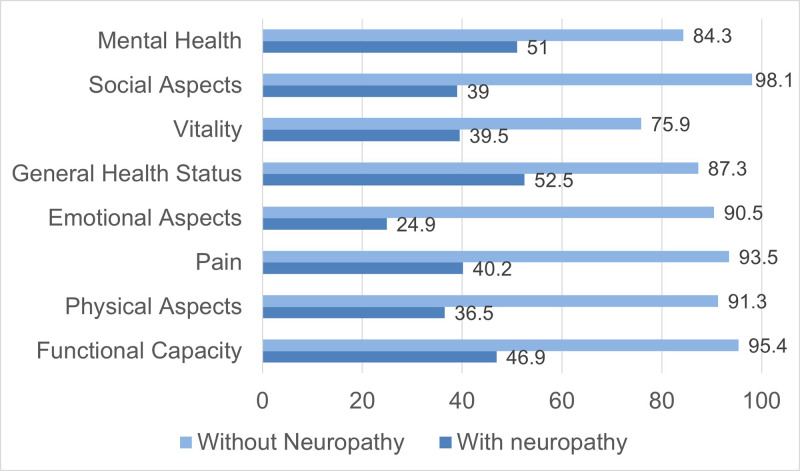
Mean of the domains of the SF-36 quality-of-life scale among HIV-positive patients with and without painful neuropathy.

Descriptive statistics for the domains of the SF-36 scale in HIV positive patients with, and without painful neuropathy are represented in Table 5 and Figure 1.

**Table 5.** Descriptive statistics for the domains of the SF-36 quality-of-life scale among HIV-positive patients with and without painful neuropathy.

SF-36 domains	HIV-positive groups	Descriptive statistics					d = effect size	Mann-Whitney test p-value
		Average	Standard deviation	Minimum	Median	Maximum		
Functional capacity	With painful neuropathy	46.9	24.1	10.0	45.0	100.0	-2.9	0.008
	No painful neuropathy	95.4	9.7	60.0	100.0	100.0		
Physical aspects	With painful neuropathy	36.5	29.5	0.0	25.0	100.0	-2.1	< 0.001
	No painful neuropathy	91.3	24.4	0.0	100.0	100.0		
Pain	With painful neuropathy	40.2	12.4	20.0	41.0	52.0	-4.2	< 0.001
	No painful neuropathy	93.5	12.9	51.0	100.0	100.0		
Emotional aspects	With painful neuropathy	24.9	32.4	0.0	25.0	100.0	-2.5	< 0.001
	No painful neuropathy	90.5	22.0	0.0	100.0	100.0		
General health status	With painful neuropathy	52.5	23.8	27.0	42.0	92.0	-1.9	< 0.001
	No painful neuropathy	87.3	14.2	40.0	89.5	100.0		
Vitality	With painful neuropathy	39.5	21.8	15.0	32.5	90.0	-1.5	< 0.001
	No painful neuropathy	75.9	26.6	0.0	87.5	100.0		
Social aspects	With painful neuropathy	39.0	26.5	12.0	25.0	100.0	-3.5	< 0.001
	No painful neuropathy	98.1	6.7	75.0	100.0	100.0		
Mental health	With painful neuropathy	51.0	20.3	24.0	42.0	92.0	-1.8	< 0.001
	No painful neuropathy	84.3	16.9	32.0	91.0	100.0		

Source: HUGG HIV/AIDS immunology outpatient clinic.

The domains that most differentiated the groups were: pain, social aspects, functional capacity, emotional aspects and physical aspects. Vitality was the domain that showed the least difference between the groups.

### Correlation between numerical variables and the SF-36 domains among patients with painful neuropathy

The greater the number of years of schooling was, the smaller the impact of the domains of emotional aspects, general health, vitality and mental health was on the quality of life of patients with painful neuropathy. In addition, the higher the income was, the lower the impact of pain, general health, vitality and mental health was on quality of life. The number of clinical manifestations related to HIV showed a negative correlation with general health status. The CD4 value showed a positive correlation with the scores for physical aspects, emotional aspects, vitality and mental health.

### Description of the correlation between categorical variables and the SF-36 scale domains

• Sex: there was no statistically significant difference between the sexes, for any of the domains of the SF-36 scale. However, all domains showed better averages for males. 

• Practicing of physical activity: Practicing of physical activity improved the quality of life of patients with neuropathic pain, especially in the functional capacity domain. 

• History of depression: Although there was no statistically significant difference between the groups, presence of this comorbidity had a significant negative influence on the following domains in the group with pain (taking into account the difference between the means): pain, general state of health, vitality and mental health. 

• Viral load: Only the physical aspects domain showed a statistically significant difference between patients who had detectable VL and those with undetectable VL, in the group with neuropathic pain.

## DISCUSSION

Thirty-seven years after the discovery of the HIV virus, much progress has been made in combating the disease caused by this virus, which without treatment is devastating. However, even today, AIDS is a public health problem that can have a major impact on morbidity among patients who live with it^13^.

Out of the sociodemographic variables studied, only the level of education showed a statistically significant difference between the groups. In an evaluation on HIV-positive patients with neuropathy, Kabongo et al., 2016, had already identified that lower educational levels correlated with worse symptoms and quality-of-life scores^14^.

Although not statistically significant, it was noted that, in relation to age, 79.2% of the patients in the group with neuropathy were older than 40 years. In this group, the patients were predominantly between 40 and 49 years old because of the inclusion and exclusion criteria. Data on increasing age as an independent risk factor for neuropathy among patients with HIV have been previously reported^15^ ^-^ ^17^. Watters et al., 2004, in a study that evaluated HIV-associated DSP patients who were older than 50 years, observed that the thin sensory fibers became less tolerant to cumulative neuropathic effects with increasing age. In addition, patients with a longer life span were unable to regulate the neuroprotective astroglial response^18^ ^,^ ^19^. Among the variables not related to HIV, the only one that showed a statistically significant difference was BMI, which was higher in the group with neuropathy. Tumusiime et at., 2014, mentioned in their study that weight gain would be related to an increased risk of developing neuropathy^20^.

Analysis on the HIV-related variables described in Table 3 showed that only the length of time with the diagnosis of HIV infection was statistically significant, such that it was higher in the group with neuropathy. Another variable that deserves to be highlighted, considering the percentage data for each group, is the length of use of ART. According to Robinson-Papp et al., 2013, and Tumusiime et al., 2014, the longer the time since the diagnosis of HIV infection, and the longer the use of ART are, the greater the risk of developing peripheral neuropathy is^20^ ^,^ ^21^. The length of use of ART was greater than 10 years for more than 54% of the patients with neuropathic pain, whereas in the group without this symptom, 65% had less than 9 years of use of ART.

Regarding CD4 and VL, in this study there was no statistically significant correlation between these variables and the presence of neuropathy. This information is in agreement with Keltner et al., 2014^22^, and Navis et al., 2018^23^.

Patients with and without neuropathic pain differ greatly with regard to almost all aspects of quality of life assessed by the SF-36 scale. Data relating to quality of life and neuropathic symptoms have already been reported from other recent studies such as Phillips et al., 2014, and Kaku and Simpson, 2014^19^ ^,^ ^15^. The findings from our study are in agreement with the results found by Phillips et al., 2014^19^, who evaluated HIV-positive patients with and without painful neuropathy using the SF-36 scale. According to these authors, the domains that showed the greatest difference between the groups were functional capacity, physical aspects, vitality and social aspects. In addition to these, in our study, the domain of emotional aspects also showed an important difference.

There were statistically significant positive correlations between the number of years of schooling, family income and BMI, and several domains of the SF-36 scale in both groups (Table 2). The age variable did not show any statistical difference. Despite this, we observed that this variable showed a negative correlation in all domains of the SF-36 scale, from which it can be inferred that increasing age may contribute to worse quality of life. Cherry et al., 2009, reported that increasing age would be related to a higher risk of developing neuropathy among patients using Stavudine^17^. Hays et al., 2000, correlated increasing age with worsening quality of life, especially when the physical aspects were evaluated^24^.

In evaluating the numerical variables related to HIV among patients with painful neuropathy and among the controls, it was observed that longer times with the diagnosis of HIV infection were correlated with worse quality-of-life scores in all the domains evaluated, and in both groups. This result is in agreement with the study by Miners et al., 2014^9^. Additionally, in our study, patients with neuropathic pain had had their diagnoses of HIV infection for longer times than the control group, and this difference was statistically significant.

The CD4 value had a positive impact on quality of life. The higher the CD4 values were, the higher the scores in all the domains assessed also were. There was a greater number of statistically significant correlations in the group with neuropathy. In this group, the correlation between CD4 and the domains of physical aspects, emotional aspects, vitality and mental health presented p values < 0.05. In the group without neuropathy, only the domain of social aspects showed a statistically significant association in relation to CD4. Thus, maintenance of adequate CD4 levels has a positive impact on several aspects of the quality of life of patients with neuropathic pain. In the literature, the data correlating CD4 and quality of life are inconsistent^25^. However, our data are concordant with those of Duncan et al., 2005, and Briongos et al., 2011^26^ ^,^ ^27^.

Practicing of physical activity had an important impact on quality of life, especially in the group with neuropathic pain. In this group, there was a statistically significant difference in the mean for the domain of functional capacity, such that it was higher among patients who were practicing physical activity. In the group without pain, no domain presented statistically significant p-values and the difference in the mean value, between those who were practicing physical activity and those who were not, was much smaller than in the preceding group. According to O'Brien et al., 2008, and O'Brien et al., 2016, aerobic physical exercise or a combination of aerobic and resistance exercises at least three times a week, and for at least 20 minutes, improved the quality of life of adult HIV patients^28^ ^,^ ^29^. According to Maharaj and Yakasai, 2018, who evaluated the influence of physical activity among HIV-positive patients with neuropathic pain, a rehabilitation program with physical exercise helps to control neuropathic pain^30^.

Jin et al., 2014, and Mannheimer et al., 2005, observed that patients with undetectable VL and better adherence to ART had better quality of life^25^ ^,^ ^31^. Our study adds that, in addition to better HIV control, prevention of risk factors for neuropathic pain helps to improve morbidity given that even patients with undetectable VL had worse quality-of-life scores when neuropathic symptoms were present.

In conclusion, the quality of life of HIV-positive patients was worse in all domains of the SF-36 scale. The domains that showed the greatest difference were pain, social aspects, functional capacity, emotional aspects, physical aspects and general health status. In assessing differences in non-HIV-related variables between groups, most patients with painful neuropathy were found to have higher BMI (overweight). Regarding the variables related to HIV, the length of time with the diagnosis of HIV infection, presence of detectable VL, use of ART and number of clinical manifestations were higher in the group with painful neuropathy. 

In evaluating the influence of variables associated with HIV on the scores of the quality-of-life scale of patients with painful neuropathy, we found that:

- Greater numbers of clinical manifestations correlated with worse general health.
- Increased CD4 levels had a positive impact on social aspects, emotional aspects, vitality and mental health.
- Detectable VL had a significant negative influence on physical aspects.

Regarding the impact of variables not related to HIV on the quality of life of this same group, we found that:

- Higher levels of education were correlated with less impact of pain on quality of life.
- Individuals who had higher family income had better general states of health, vitality and mental health.
